# Two specific drugs, BMS-345541 and purvalanol A induce apoptosis of HTLV-1 infected cells through inhibition of the NF-kappaB and cell cycle pathways

**DOI:** 10.1186/1742-6405-5-12

**Published:** 2008-06-10

**Authors:** Emmanuel Agbottah, Wen-I Yeh, Reem Berro, Zachary Klase, Caitlin Pedati, Kyleen Kehn-Hall, Weilin Wu, Fatah Kashanchi

**Affiliations:** 1 Department of Microbiology and tropical Medicine and Department of Biochemistry and Molecular Biology, The George Washington University School of Medicine, Washington, District of Columbia 20037, USA; 2 Department of Microbiology, Institute for Proteomics Technology and Applications, The George Washington University, Washington, District of Columbia 20037, USA; 3 The Institute for Genomic Research, TIGR, Rockville, Maryland 20850, USA

## Abstract

Human T-cell leukemia virus type-1 (HTLV-1) induces adult T-cell leukemia/lymphoma (ATL/L), a fatal lymphoproliferative disorder, and HTLV-1-associated myelopathy/tropical spastic paraparesis (HAM/TSP), a chronic progressive disease of the central nervous system after a long period of latent infection. Although the mechanism of transformation and leukemogenesis is not fully elucidated, there is evidence to suggest that the viral oncoprotein Tax plays a crucial role in these processes through the regulation of several pathways including NF-κB and the cell cycle pathways. The observation that NF-κB, which is strongly induced by Tax, is indispensable for the maintenance of the malignant phenotype of HTLV-1 by regulating the expression of various genes involved in cell cycle regulation and inhibition of apoptosis provides a possible molecular target for these infected cells. To develop potential new therapeutic strategies for HTLV-1 infected cells, in this present study, we initially screened a battery of NF-κB and CDK inhibitors (total of 35 compounds) to examine their effects on the growth and survival of infected T-cell lines. Two drugs namely BMS-345541 and Purvalanol A exhibited higher levels of growth inhibition and apoptosis in infected cell as compared to uninfected cells. BMS-345541 inhibited IKKβ kinase activity from HTLV-1 infected cells with an IC_50 _(the 50% of inhibitory concentration) value of 50 nM compared to 500 nM from control cells as measured by *in vitro *kinase assays. The effects of Purvalanol A were associated with suppression of CDK2/cyclin E complex activity as previously shown by us. Combination of both BMS-345541 and Purvalanol A showed a reduced level of HTLV-1 p19 Gag production in cell culture. The apparent apoptosis in these infected cells were associated with increased caspase-3 activity and PARP cleavage. The potent and selective apoptotic effects of these drugs suggest that both BMS-345541 and Purvalanol A, which target both NF-κB and CDK complex and the G1/S border, might be promising new agents in the treatment of these infected patients.

## Background

Human T-cell leukemia virus type 1 (HTLV-1) is associated with aggressive adult T-cell leukemia (ATL) and HTLV-1-associated myelopathy/tropical spastic paraparesis (HAM/TSP) [1]. ATL arises after a long latent period of over 50 years and involves with a multi-step mechanism of tumorigenesis [2]. The transforming ability of HTLV-1 is primarily due to the viral oncoprotein, Tax [3]. Tax not only transactivates viral genes by binding to CREB but also activates cellular transcriptional factors including nuclear factor kappa B (NF-κB), cyclic AMP responsive element, CREB-binding protein, TATA-binding protein and TFIIA [4-14]. Acute ATL is an aggressive leukemia with a median survival of only 6 months and a projected 4-year survival of about 5% [2].

NF-κB transcription factor plays a crucial roles in tumorigenesis and tumor development [15,16]. NF-κB transcription factor controls the expression of genes involved cell cycle regulation and apoptosis, such as *cyclin E, bcl-2, bcl-x*_*L *_, *c-IAPs, survivin*, and *XIAP *[16-18]. Vertebrate NF-κB transcription complexes can be any of a variety of homo- and heterodimers formed by the subunits p105/p50, p100/p52, c-Rel, p65 (RelA) and RelB [19]. There are multiple pathways to activate NF-κB. The two most common pathways are the canonical and the non-canonical pathways [20,21]. In the canonical pathway, proceeding the stimulation of TNF-R, the activated IκB kinase (IKK) complex containing IKKα/IKKβ/NEMO phosphorylates inhibitor of NF-κB (IκBα) [22,23]. The phosphorylated IκBα (Ser32/S36) is then ubiquitinated and degraded, which allows NF-κB (p50–p65) to enter the nucleus where it regulates the expression of specific genes [24]. In the non-canonical pathway, the IKK complex with two IKKα subunits is activated through NIK by other stimuli such as lymphotoxin β (LTβ) and CD40 ligand, and mediates the processing of NF-κB complex to p52/RelB [25,26]. This IKK complex then phosphorylates p100 at C-terminal domain and promotes the ubiquitination of p100 and the proteasomal processing of the complex to p52/RelB [27-29].

A number of reports have elucidated that the HTLV-1-infected T-cells are associated with constitutively activated NF-κB and its involvement in tumorigenesis [25,26,30,31]. Tax is known to activate NF-κB by stimulating IKK complex in both canonical and non-canonical pathways by interacting with NEMO [32-35]. Tax is also reported to directly bind to and activated NF-κB [4]. The role of various transcription factors in tumorigenesis has previously been described [36]. NF-κB and AP-1 have recently been implicated in cell survival and proliferation pathways. The NF-κB pathway is activated in ATL cells that do not express Tax, although the mechanism of activation remains unknown [37]. One of the potential mechanisms by which these cells could develop resistance to apoptosis is through the activation of NF-κB [38]. From this point of view, NF-κB has become an attractive target for therapeutic intervention. Indeed, inhibition of the NF-κB pathway by Bay 11–7082, an irreversible inhibitor of IκBα phosphorylation [25,39], by dehydroximethylepoxy-quinomicin, an inhibitor of nuclear translocation of p65, a component of NF-κB [40-42], arsenic trioxide on NF-κB [43,44]. and by bortezomib, a proteasome inhibitor [45], induced apoptosis of HTLV-I-infected T-cells and ATL cells, suggesting that inhibitors of NF-κB may be effective targets against ATL cells *in vivo*.

In addition to the regulation of NF-κB pathway, viral transactivator Tax provides some initial alternation in cell cycle progression to the proliferation of viruses. HTLV-1 and/or Tax-expressing cells have altered expression of some cell cycle-associated genes and accelerate cell cycle progression in G_1 _phase [46-50]. Tax targets cell cycle regulators such as p53, cyclin dependent kinases (CDKs) 4 and 6, cyclin D2, and CDK inhibitors p21^waf1 ^and p16^INK4A ^[51-57]. Tax expression also results in transcriptional activation of cyclin E and CDK2 complex [58-61]. In addition, the cyclin E/CDK2 kinase activity is shown to be increased in HTLV-1 infected cells [62].

Currently there is no accepted curative therapy for ATL or HAM/TSP and the conditions, at least in the ATL, often progresses to death with a median survival time of 13 months [63]. The prognosis of this aggressive stage remains poor, and death is usually due to severe infection or hypercalcemia, often associated with resistance to intensive, combined chemotherapy. Therefore, the establishment of new therapeutic strategies for HTLV-1 infected cells is deemed critical. Due to the presence of highly activated NF-κB pathway and tightly controlled cell cycle progression the infected cells rely on these two mechanisms for its survival and possibly progeny formation. In an effort to find novel inhibitors, we initially screened thirty-five inhibitors targeting these two pathways to examine their effect on cell growth. Two inhibitors BMS-345541 and Purvalanol A showed the best selectivity in inhibiting HTLV-1 infected, but not uninfected, cells. Utilizing a series of biochemical assays, we determined that BMS-345541 inhibited IKKβ activity *in vitro *and induced higher level of apoptosis in infected cells. Finally, the efficacy of combination of both BMS-345541 and Purvalanol A in inhibiting HTLV-1 infected cells was tested. Collectively, understanding the inhibition mechanism, efficiency and the combined effects of both BMS-345541 and Purvalanol A will help gain better insights and establish novel new therapeutic approaches for HTLV-1 infected patients.

## Results

### Screening of various inhibitors on HTLV-1 infected and uninfected cells

Despite its tight control in normal T cells, NF-κB is constitutively activated in both HTLV-I-transformed T-cell lines and freshly isolated ATL cells suggesting that activation of NF-κB is an important part of the oncogenic mechanism of HTLV-I. This pathologic action may largely rely on the viral transforming protein Tax, at least for many of the cell lines to date that are isolated for *in vitro *analysis and not necessarily are ATL samples, which also up-regulates the expressions and activities of cyclin E/CDK2 which is important in cell cycle transition from G_1 _to S phase.

Most importantly, IKK has been established as a cellular target of Tax and an essential component in Tax-mediated NF-κB signaling in both canonical and non-canonical pathways. Therefore, we reasoned that the specific targeting of both the NF-κB signaling and cell cycle regulators with drugs might provide better insights into how to inhibit HTLV-1 infected cells. We sought to identify the targets of a range of NF-κB and CDK inhibitors in HTLV-1 infected and uninfected cells by culturing MT-2, MT-4, C8166, c10/MJ and uninfected CEM and Jurkat T-cells (0.5 × 10^6 ^cells/well) in media with inhibitor concentrations ranging from 0, 0.01, 0.1, 1, and 10 μM. Cells were treated for 48 hours and the level of growth inhibition was estimated using trypan blue method. Results from 35 drugs that inhibit various CDKs and IKKs are shown in Table 1 where a number of drugs inhibited HTLV-1 infected cells much more efficiently than uninfected cells. Among the top two candidates that inhibited HTLV-1 infected cells were BMS-345541 (4(2'-aminoethyl)amino-1,8-dimethylimidazo(1,2-a)quinoxaline) and Purvalanol A. BMS-345541 is a selective inhibitor of IKKβ at IC_50 _of 0.3 μM and to a lesser extent an inhibitor of IKKα at IC_50 _of 4 μM [64,65]. All drugs were further tested at 10 μM concentration to effectively compare these different classes of inhibitors against one another. In Table 1, they are ranked as high, moderate, and poor inhibitors and the reported activities of these molecules against variety of CDKs and IKKs are indicated in the right-hand column. Collectively, these data indicate that initial cell based survival screening assays may be an effective tool in isolating drugs that are more selective against HTLV-1 infected cells as compared to control uninfected cells.

**Table 1:** Screening of Various CDK and NFkB/IKK Inhibitors and Related Molecules for HTLV-1 Cell Killing

Selectivity	Name		MT-2	MT-4	C8166	C10/MJ	CEM	Jurkat	Reported activities of molecules (IC50 in nM)
			Infected				Uninfected		
High	BMS-345541	(10 uM)	++++*	+++	+++	+	- **	-	IKK-1(4), IKK-2(0.3)
	Purvalanol A	(10 uM)	++++	++	++	-	-	-	CDK1(4), CDK2(70), CDK5(75)
	Indirubin-3'-monoxime	(10 uM)	+++	+++	+++	+++	++	++	CDK1(180), CDK2(250), CDK4(3330), CDK5(100)
	Indirubin-3'-monoxime-5'-Iodo	(10 uM)	+++	+++	+++	+++	++	++	CDK1(25), CDK5(20)
	9-Cyanopaullone	(10 uM)	++++	+	-	-	-	-	CDK1(24), CDK5(44)
	Aloisine A	(10 uM)	+++	+	-	-	-	-	CDK1(150), CDK2(120), CDK5(200)
	Compound 52	(10 uM)	+++	+	-	-	-	-	CDK1(340)
	Flavopiridol	(0.1 uM)	++	+	-	-	-	-	CDK9(50)
Moderate	r-Roscovitine	(10 uM)	++	-	-	-	-	-	CDK1(650), CDK2(700), CDK5(160), CDK7(500)
	Bohemine	(10 uM)	++	-	-	-	-	-	CDK1(1000)
	s-Roscovitine	(10 uM)	+	-	-	-	-	-	CDK1(650), CDK2(700), CDK5(160), CDK7(500)
	WHI-P180	(10 uM)	+	N/A	N/A	-	N/A	N/A	CDK2(1000)
	Kenpaullone	(10 uM)	+	-	-	-	-	-	CDK1(400), CDK2(680), CDK5(850)
	2,6-Diaminopurine	(10 uM)	-	+	-	-	-	-	
	Flavone	(10 uM)	+	-	-	-	-	-	CDK1(300), CDK2(100), CDK4(400), CDK7(300)
	Alsterpaullone	(10 uM)	++++	++	+++	+	++	++	CDK1(35), CDK2(15), CDK5(40)
	CGP 74514A	(10 uM)	++++	++	+++	+	++	++	CDK1(25)
	BAY 11-7085	(10 uM)	+	++++	+++	N/A	++++	++++	
	BAY 11-7082	(10 uM)	+	++++	+++	N/A	++++	++++	IkBa(10000)
	CAPE	(10 uM)	+	+++	-	N/A	++++	++++	
	Diethylmaleate	(10 uM)	+	+++	-	N/A	++++	++++	
	Parthenolide	(10 uM)	+	+++	+++	N/A	++++	++++	
	Pyrrolidinedithiocarbamic acid	(10 uM)	-	+++	+++	N/A	++++	++++	
	Wedelolactone	(10 uM)	+	++	-	N/A	++	++	
Poor	6-Benzyloxypurine	(10 uM)	-	-	-	-	-	-	
	5-amino alicylic acid	(10 uM)	-	-	-	N/A	-	-	
	2,6-Dichloropurine	(10 uM)	-	-	-	-	-	-	
	6-Dimethylaminopurine	(10 uM)	-	-	-	-	-	-	CDC
	Indirubin-3'-monoxime-5'-sulphonic acid	(10 uM)	-	-	-	-	-	-	CDK1(5), CDK5(7)
	Iso-olomoucine	(10 uM)	-	-	-	-	-	-	CDK1(>500,000), CDK4,5(>1,000,000)
	N-6-(Δ2-Isopentyl)-adenine	(10 uM)	-	-	-	-	-	-	CDK1,2,5(>50,000)
	Olomoucine	(10 uM)	-	-	-	-	-	-	CDK1,2(7000), CDK5(3000)
	Olomoucine, N9-isopropyl	(10 uM)	-	-	-	-	-	-	CDK1(2000)
	SC-514	(10 uM)	-	-	-	-	-	-	IKK-1(>200), IKK-2(11.2)
	QNZ	(10 uM)	-	-	-	N/A	++	+	

	* positive sign indicates level of cell inhibition		Inhibition	Percentage					
	** negative sign indicates no cell inhibition		-	1–5%					
			+	25%					
			++	50%					
			+++	75%					
			++++	90%					

### Effect of BMS-345541 on IKKβ in infected and uninfected cells

**Figure 1:**
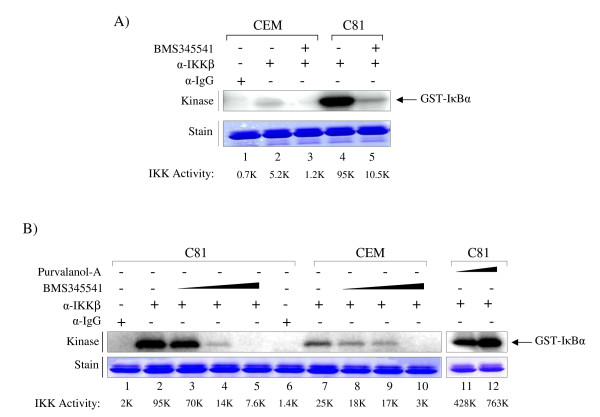
**BMS-345541 inhibition of IKKβ in HTLV-1 infected cell**. **A) **BMS-345541 reduced IKKβ activity in C8166 cells. Equal amount (1 mg) of cytoplasmic proteins was immunoprecipitated with anti-IKKβ antibody and mixed with 1 μM BMS-345541. The IKKβ activities were examined by *in vitro *kinase assay using GST-IκBα as a substrate. The [γ-^32^P]-labeled IκB-α protein was visualized by autoradiography. The IKKβ activities were quantitated by ImageQuant software. The bottom panel shows a commassie blue staining of GST-IκBα to show equal amount of substrate in each reaction. **B) **BMS-345541 inhibited IKKβ activity in C8166 cells in dose-dependent manner; however, Purvalanol A had no effect on IKKβ. Kinase assay were performed as described above using 0.01, 0.1, and 1 μM of BMS-345541 and 1, 10 μM of Purvalanol A. The stained gel below is a representative of the kinase reaction.

We next focused our attention on BMS-345541 and asked whether this drug could inhibit the IKKβ kinase activity on its substrate IκBα. We immunoprecipitated (IP) IKKβ from both CEM (uninfected) and C8166 (infected) cells and used them in an in vitro kinase assays in the presence or absence of BMS-345541 (1.0 μM). Results are shown in Figure 1A where C8166 cells had far stronger IKKβ kinase activity as compared to CEM cells (compare lanes 2 to 4). Active kinases that were incubated with BMS-345541 showed a reduction of activity from both infected and uninfected cell extracts. However, the inhibition was much more dramatic with kinases isolated from HTLV-1 infected cells. We next titrated various levels of BMS-345541 for both kinases in our in vitro assay. Results are shown in Panel B where 0.01, 0.1, and 1.0 μM of BMS-345541 were used for a complete range of titrations. Interestingly, at 0.1 μM there was a significant reduction in the kinase activity from infected cells (lane 4 compared to lane 9). A control drug, Purvalanol A, which is a CDK inhibitor, did not inhibit the IKKβ kinase activity obtained from infected cells. Collectively, these results indicate that IKKβ from infected cells is much more sensitive to BMS-345541 as compared to IKKβ from uninfected cells.

### Induction of apoptosis in HTLV-1 infected cells by BMS-345541

**Figure 2:**
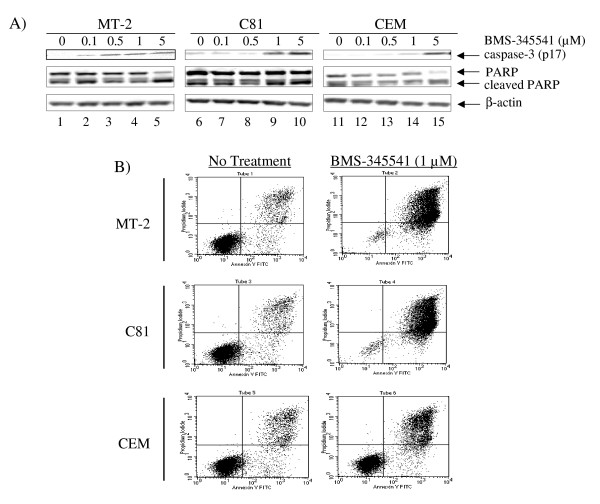
**BMS-345541 induction of apoptosis in C8166**. A) BMS-345541 induced caspase-3 and PARP cleavage C8166. MT-2, C8166, and CEM cells were treated with BMS-345541 at 0.1, 0.5, 1, and 5 μM for 48 hr. Total cell extracts were subjected to Western blot analysis for caspase-3 and PARP. β-actin Western blot was used as internal control. The results of caspase-3 were quantitated and normalized with β-actin. The ratio of c/un PARP was calculated by dividing cleaved PARP to un-cleaved PARP (data not shown). **B) **Detection of apoptosis through annexin V and PI staining. Cells were washed three times in PBS and re-suspended in binding buffer, stained with annexin V-FITC and PI for 15 minutes at room temperature. Analysis was performed on a BD FacsCalibur flow cytometer.

Resistance to cell apoptosis is one of the mechanisms that is important and is also required for the immortalization of T cells [63,66]. NF-κB signaling pathway is the survival pathway activated by HTLV-1 in order to keep the host cell active [67,68]. BMS-345541 targets IKKβ subunit which is responsible for activation of the NF-κB pathway [64,65]. To determine whether BMS-345541 can inhibit NF-κB pathway and induce apoptosis in HTLV-1 infected cells, we analyzed the level of apoptotic markers such as caspase-3 and PARP in both infected and uninfected cells. Caspase-3 is a member of cysteine protease and plays a key role in apoptosis [69]. When apoptosis is activated, the inactive pro-caspase-3 is processed into active large (17 kD) and small (12 kD) subunits [70]. PARP, poly(ADP-ribose) polymerase, is also an apoptosis marker that is cleaved from precursor form (116 kD) into active form (85 kD) by active caspase-3 during apoptosis [71-73]. Results in Figure 2A are Western blots that show titration of BMS-345541 in two infected and one uninfected cells. Samples were treated for 48 hours and extracts were made for Western blotting. The top panel shows the caspase Western and a gradual increase of p17 form in MT-2 cells as well as C8166 cells in concentrations between 0.5 and 1.0 μM. There was no change in the actin levels in any of the samples treated. Panel B shows the results of the Annexin V staining where live cells are represented at the bottom right corner box in each panel. All three samples were treated with 0.1 μM of BMS-345541 and stained for the presence of live and apoptotic cells. Interestingly both MT-2 and C8166 cells showed presence of few live cells as compared to CEM cells when treated with BMS-345541. Collectively, these data indicate that low concentrations of IKKβ inhibitor can apoptosis HTLV-1 cells much more efficiently as compared to uninfected cells.

### Effect of BMS-345541 on inhibition of IκB and p65 phosphorylation in vivo

**Figure 3:**
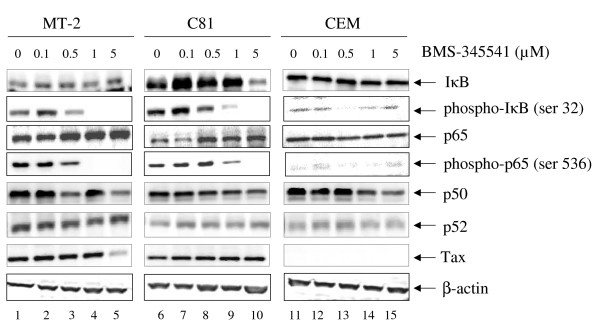
**Effect of BMS-345541 on inhibition of IκB and p65 phosphorylation in vivo**. MT-2, C8166, and CEM cells were treated with BMS-345541 at 0.1, 0.5, 1, and 5 μM for 48 hr. Total cell extracts were collected and subjected to Western blot analysis using anti-IκB, phospho IκB (ser 32), p65, phospho p65 (ser 536), p50, p52, Tax and actin. Twenty five microgram of each extract was used to separate on a 4–20% SDS/PAGE. Levels of total IκB and p65 did not change between cell types, however there was a dramatic increase of phosphor-IκB and phosphor-p65 in HTLV-1 infected cells and their suppression by BMS-345541 which inhibits IKKβ activity in vivo.

We subsequently asked if IκB or p65 levels could be altered in drug treated infected and uninfected cells. We therefore Western blotted our drug treated cells with antibodies against IκB, phospho IκB (ser 32), p65, phospho p65 (ser 536), p50, p52, Tax and actin. Both ser 32 of IκB and ser 536 of p65 are phosphorylated by IKKβ in vivo. Results of such an experiment are shown in Figure 3 where IκB levels essentially stayed the same in all three cell lines except for a drop in C8166 cells at 5.0 μM. We have previously observed that cells, irrespective of infection, treated with BMS-345541 at higher does (i.e., 10.0 μM) are toxic and show non-specific activation of apoptotic machinery (data not shown). There was also no change in levels of p65 although a slight increase in C8166 cells was observed at higher concentrations. A more interesting set of results were observed with phosphor-IκB and phosphor-p65 blots. MT-2 cells treated with BMS-345541 showed a reduction of both phosphor-IκB and phosphor-p65 levels at 0.5 μM. Similar results were also seen in C8166 cells. Very little phosphor-IκB and phosphor-p65 were observed in CEM cells (or other control Jurkat cells, data not shown). P50, p52 levels were unchanged with various drug concentrations and Tax levels were not decreased at 0.5 or 1.0 μM concentration of the drug. No changes were seen in the actin levels in any of the treated cells. Collectively, these results indicate that inhibition of IKKβ in HTLV-1 infected cells by BMS-345541 affects phosphorylation of both IκB and p65 molecules, both of which may be the hallmarks of NF-κB activation in HTLV-1 infected cells.

### Inhibition of cyclin/CDK complexes by Purvalanol A

**Figure 4:**
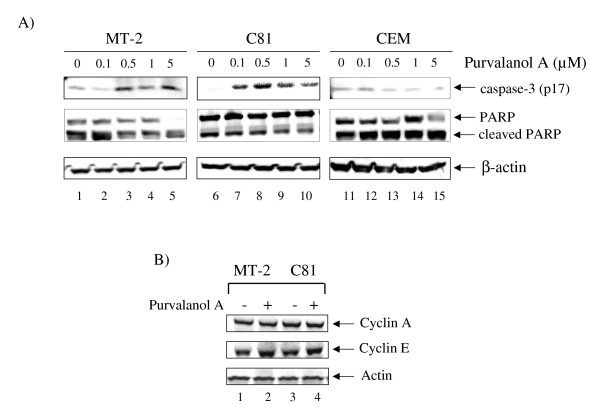
**Purvalanol A induction of apoptosis in MT-2**. **A) **Purvalanol A induced caspase-3 and PARP cleavage in MT-2 and C8166 cells. MT-2, C8166, and CEM were treated with Purvalanol A at various 0.1, 0.5, 1, and 5 μM for 48 hr. After 48 hr of treatment, total cell extracts were collected and subjected to Western blot analysis of caspase-3 and PARP. Westeron blot of β-actin was used as an internal control. **B) **Twenty five microgram of each Purvalanol A treated (5 μM for 48 hr) extracts from both MT-2 and C8166 cells were also used for western blot against cyclin A, E and actin.

**Figure 5:**
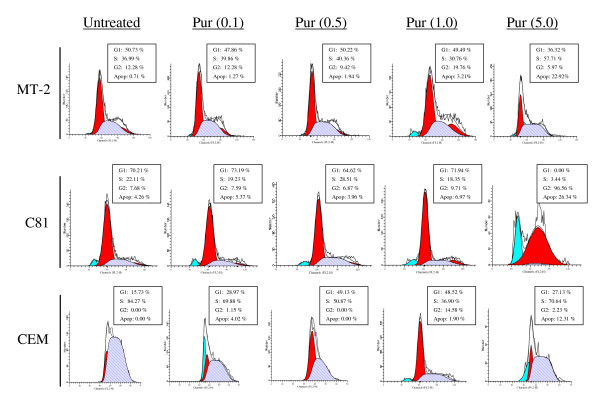
**Cell cycle analysis of cells treated with or without drugs**. For fluorescence-activated cell sorting (FACS) analysis, both untreated and Purvalanol A treated CEM, MT-2 and C8166 cells (0.1 – 5.0 μM) were stained with a mixture of propidium iodide buffer followed by cell sorting analysis. The acquired FACS data were analyzed by ModFit LT software (Verity Software House, Inc.).

We have previously shown that cyclin E/CDK2 kinase activity is de-regulated in HTLV-1 infected cells and these cells are especially susceptible to Purvalanol A treatment [62]. Moreover, Purvalanol A, which is a purine analog that competes with the ATP binding site in CDKs, has been shown to inhibit cyclin E/CDK2 and cyclin A/CDK2 kinase activities with an IC_50 _of 0.035 and 0.07 μM, respectively [74-77]. We therefore treated both infected and uninfected cells for 48 hours with Purvalanol A and Western blotted for caspase-3 and PARP molecules. Results in Figure 4A show that the caspase-3 p17 molecule was present in infected cells treated with 0.1 and 0.5 μM of Purvalanol A (lanes 3 and 7). This was important since Purvalanol A did not significantly activate caspase-3 in CEM (lanes 11–15) or Jurkat cells (data not shown). There were no changes in actin (bottom panel), cyclin E, or cyclin A expression levels when treated with Purvalanol A (Panel B). Therefore Purvalanol A treatment induces caspase-3 activation in HTLV-1 infected, and not in uninfected cells. This is consistent with our previous results where Purvalanol A treatment of infected cells inhibited cyclin E/CDK2 complex activity in HTLV-1 infected cells, inhibited transcription of the LTR promoter and promoted apoptosis [62]. Along these lines, we also assayed for changes in cell cycle progression and apoptosis in these cells using FACS analysis. Results in Figure 5 show the titration of Purvalanol A for all three cell types. Interestingly, significant apoptosis appeared in infected cells treated at 1.0 and 5.0 μM concentrations.

### Inhibition of viral replication using both drugs

**Figure 6:**
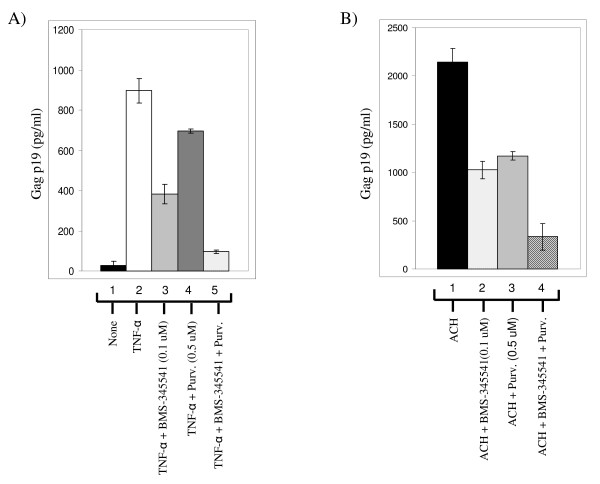
**Double drugs treatment results in lower p19 Gag levels in HTLV-1 infected cells**. **A) **MT-2 cells (HTLV-1 infected) were treated with TNF-α (10 ng/ml) for 2 h, washed, and subsequently treated with a specific NF-kB or CDK inhibitor. Activated cells were subsequently treated them with BMS-345541 alone (0.1 μM), Purvalanol A alone (0.5 μM), or a combination of both drugs. Samples were collected after 7 days and used for detection of p19 Gag using ELISA. **B) **Log phase 293 cells were transfected with ACH.pcTax (wild type HTLV-1 clone, generous gift of Dr. Lee Ratner, Washington University) using electroporation method. After transfection, the cells were cultured in complete medium and culture supernatants were collected at 4 days post-transfection, and virus particle production was monitored by p19 ELISA. Drug treatments (as in panle A) were 6 hrs after transfection of the 293 cells for a total of 150 hrs.

We next decided to use both drugs in a viral replication assay in MT-2 cells. MT-2 cells normally produce low levels of infectious HTLV-1 virions that could be detected in the supernatant using p19 gag ELISA. However, treatment of these cells with TNF can produce at least 1–2 log more virus that is shed into the supernatant. We therefore treated MT-2 cells with TNF for 2 hours and subsequently treated them with BMS-345541 alone (0.1 μM), Purvalanol A alone (0.5 μM), or a combination of both drugs. Results in Figure 6A show that, as compared to untreated cells, TNF treatment induced high amounts of p19 gag in the supernatant (lanes 1 and 2). Both drugs alone reduced p19 levels to some degree however; the best inhibition was seen with the combination of both drugs where NF-κB and CDK pathways were targeted in these cells. Similar results were also obtained in 293 cells transfected with ACH full-length infectious clone, where a combination of both drugs inhibited p19 expression as compared to when treated with one drug alone (Panel B). Collectively, these results imply that low concentrations of NF-κB and CDK inhibitors that normally do not cause cell death in uninfected cells are effective inhibitors against HTLV-1 infected cells.

## Discussion

In contrast with the latest progress in the understanding of HTLV-1 infection, its pathogenesis and its mechanism of action, more progress in developing therapies for these infected cells is needed. There has been only very limited improvement in the prognosis of virally associated diseases (ATL and HAM/TSP) during the past several years. However few well established pathways including NF-κB and cell cycle progression have been shown to be tightly regulated in HTLV-1 and Tax expressing cells and therefore providing viable targets for treatment [51,78,79]. Along these lines, we searched various inhibitors targeting these two pathways using published literature and our own search using few small libraries of compounds tested here. We selected inhibitors with low-high IC_50 _in various cell types and identified their cell growth inhibition efficiencies in HTLV-1 infected and uninfected cells. Results in Table 1 clearly show that there are various compounds that specifically target HTLV-1 (and Tax) producing cells. Many of these compounds have known targets and more importantly are not inhibitors of other viruses including HIV-1 (more then 78% have different IC50 in HIV-1 infected cells, data not shown). Furthermore, the inhibitors in high selectivity group showed higher inhibition efficiency in MT-2 cells which normally produces some level of full length infectious HTLV-1 particles in the absence of any inducer. Therefore, it is interesting to note that these inhibitors not only had specificity to inhibit Tax expressing cells but also showed better growth inhibition toward infected cells that produce high titer virus.

In high selectivity group, BMS-345541 and Purvalanol A demonstrated the best selectivity to block growth of all HTLV-1 infected cells and no blockage to control cells in these concentrations (Table 1). Indirubin-3'-monoxime and 5'-Indo-indirubin-3'-monoxime inhibited growth of infected cells and also inhibited control cells. 9-Cyanopaullone, Aloisine A, Compound 52, and Flavopiridol showed less growth inhibition in inhibiting two out of four infected cell lines. Consequently, we decided to focus and study the mechanism of BMS-345541 and Purvalanol A inhibition in HTLV-1 infected cells.

**Figure 7:**
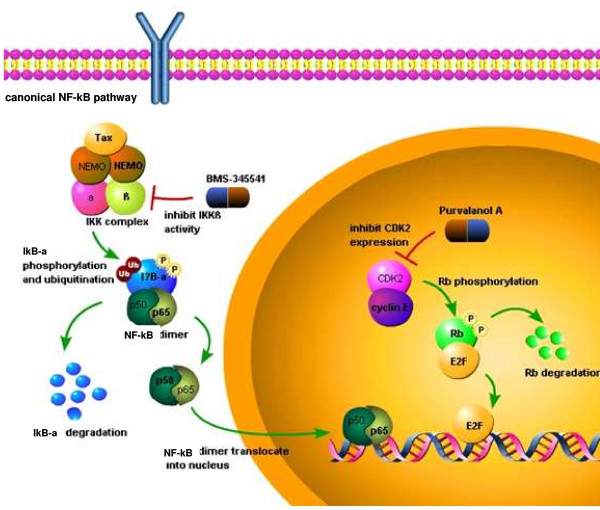
**Inhibition mechanism of BMS-345541 and Purvalanol A in HTLV-1 infected cells results in blocking canonical NF-κB signaling pathway and cell cycle progression**. In the absence of drug, hyperactive IKK complex phosphorylates IκB-α resulting in IκB-α degradation and p65/p50 translocation. The genes transcribed by p65/50 include anti-apoptotic genes which are responsible for survival of virus infected cells. In the presence of BMS-345541, the activity of IKK complex is inhibited which results in decreased IκB-α phosphorylation, therefore p65/p50 are kept in cytoplasm. Hence, the expression of anti-apoptotic proteins are decreased which make HTLV-1 infected cells more susceptible and sensitive to the action of the drug. Without drugs, cyclin E/CDK2 phosphorylates Rb and induces Rb degradation. The free E2F then transcribes genes which are necessary for G1/S transition. However, Purvalanol A inhibits CDK2 (a non-essential protein in the life cycle of a cell) activity, as previously shown by us, which results in decreased Rb phsophorylation and inactivated E2F. Therefore, the infected cells may be blocked at the G_1 _checkpoint and simultaneously have lower viral expression.

In this study, we showed that BMS-345541 inhibited IKKβ kinase activity from HTLV-1 infected cell. IKKβ subunits associating with canonical pathway is responsible for activating NF-κB by phosphorylating IκBα. Furthermore, BMS-345541 induced higher level of apoptosis in C8166 and other cells (data not shown). Therefore, we speculated that BMS-345541 suppressed IKKβ and further blocked NF-κB signaling pathway, the survival pathway, to induce apoptosis. As illustrated in our model, in the presence of BMS-345541, the level of unphosphorylated IκBα is expected to increase and keep NF-κB dimmers in cytoplasm and block its transcriptional ability (Figure 7). In addition, IKKβ activity in C8166 was dramatically down-regulated by BMS-345541 with an IC_50 _at 0.05 μM in a dose-dependent manner, whereas the IC_50 _in CEM cell was at 0.5 μM. The HTLV-1 infected cell was at least 10 times more sensitive to BMS-345541 than control cells. This critical difference is thought to be the related to the NF-κB pathway in HTLV-1 infected cell. NF-κB is tightly controlled in normal T-cells; however, HTLV-1 control of the host cells depends on constitutively activated NF-κB for quelling apoptosis. Inhibition of NF-κB in HTLV-1 infected cell is tantamount to blocking the significant survival pathway.

In infected patients, dysregulation of cell cycle regulatory proteins is considered to promote cell cycle progression and overcome cellular checkpoints. Tax activates the expression of cyclin D2, cyclin E, CDK2, and CDK4 and the kinase activity of cyclin E/CDK2 which accelerates G_1 _/S transition and promotes passage through the restriction point immediately [2,51]. Furthermore, it has been shown that other viruses such as Epstein-Barr virus (EBV) also accelerates viral replication by activating S-phase promoting CDKs such as cyclin E/CDK2 and cyclin A/CDK2 and consequently accumulating hyperphosphorylated non-functional Rb [80]. In this study, we identified the CDK inhibitor with the best specificity to ATL cells to be Purvalanol A (Table 1). This drug showed induction of apoptosis as evident from increased caspase 3 activity. Purvalanol A was previously shown by us to effect the *in vivo *transcription of HTLV-1 promoter and inhibit viral replication and cell growth by MTT assay [81].

An important advance in the treatment of ATL was reported in two preliminary phase II studies with the combination of an anti-retroviral agent zidovudine (AZT) and interferon-α (IFN-α) in previously untreated, as well as in relapsed acute ATL and ATL lymphoma [82-84]. The phase II study showed a high response rate which has never been previously reached with any chemotherapy regimen [85]. Dual drugs treatment with arsenic trioxide and IFN-α in ATL patients also had significant inhibition and specificity in phase II trial [86]. Arsenic trioxide targets the NF-κB pathway by stabilizing IκB-α and IκB-ε [44]. The combination drug treatment induced proteasomal degradation of Tax and resulted in the reversal of NF-κB transcription factor activation [87]. Therefore, we utilized a combined treatment of HTLV-1 infected cells with BMS-345541 and Purvalanol A. We performed similar experiments in MT-2 cells that can produce high amounts of virus after TNF treatment. Interestingly, combination of both drugs at low concentration inhibited viral production without having any toxic effects (in either infected or uninfected cells). Although it should also be noted that our results don't show if Purvalanol A and BMS-345541 prevent cells from HTLV-1 infection and whether possible receptor(s) of HTLV-1 infection are altered when using these drugs. Collectively, combination of two drugs that can inhibit both NF-κB and CDK machineries in HTLV-1 "hyper-active" cells seem to be a viable option in inhibiting infection. Future experiments are in progress to develop second and third generation drugs, as well as their effect in fresh ATL samples and inhibition in mouse models.

## Conclusion

Recently, unique therapeutic approaches targeting molecules and/or mechanisms involved in the pathogenesis of HTLV-1 have been explored, and some have produced encouraging results that might lead to breakthrough therapies. In this study, we have demonstrated that two drugs (BMS-345541 and Purvalanol A) out of thirty-five drugs studied that target NF-κB or CDK pathways had the best specificity in inhibiting the growth of HTLV-1 infected but not uninfected cells. The effect of BMS-345541 is through the inhibition of IKKβ kinase activity resulting in dephosphorylation of IκBα and inactivation of NF-κB pathway. The specificity of BMS-345541 with IC_50 _of 50 nM in HTLV-1 infected cell compared to IC_50 _of 500 nM in uninfected cell therefore renders the infected cells 10 times more sensitive to the drug than uninfected cell. The other inhibitor, Purvalanol A induced higher level of inhibition in MT-2 cells and the mechanism was previously shown by us to be associated with inhibition of functional cyclin E/CDK2 complexes. Combination of these two inhibitors induced even higher level of p19 Gag expression in infected cells. Therefore, treatment of HTLV-1 infected cells with either BMS-345541, Purvalanol A or a combination of these two drugs hold promising leads in treatment of infected cells.

## Methods

### Cell lines and reagents

MT-2, MT-4, C8166, and C10/MJ were all obtained from NIH AIDS Research & Reference Reagent Program. They are all HTLV-1 infected cell lines and some including C8166 contain defective viruses but still express Tax. MT-2 cells carry multiple copies of the HTLV-1 cosmopolitan subtype and normally produce some full length infectious HTLV-1 particles in the absence of any inducer [157]. MT-4 cells are established from the human T cells isolated from a patient with adult T-cell leukemia. CEM and Jurkat cells are the uninfected control T lymphocyte cell lines. All cell lines were cultured at 37°C up to 1 × 10^5 ^cells per ml in RPMI 1640 medium containing fetal bovine serum (10%), streptomycin, penicillin antibiotics (1%) and L-Glutamine (1%) (Gibco/BRL). The CDK inhibitors used were: Aloisine A (270-385-M001), Alsterpaullone (270-275-M001), Bohemine (270-390-M001), CGP74514A (270-391-M001), Compound 52 (270-248-M001), 9-cyanopaullone (270-282-M001), 6-dimethylaminopurine (480-050-M100), indirubin-3'-monoxime (270-271-M001), 5-iodo-indirubin-3'-monoxime (270-424-M001), N-6-(Δ2-Isopentenyl)-adenine (350-034-M100), Kenpaullone (270-274-M001), Olomoucine (350-013-M005), N9-isopropylolomoucine (270-397-M001), Purvalanol A (270-246-M001), (R)-Roscovitine (350-251-M001), (S)-Roscovitine (350-293-M001) were purchased from Alexis Inc. and 6-benzyloxypurine (387606), 2,6-diaminopurine (247847), 2,6-dichloropurine (D73103), Flavone (F2003) were purchase from Sigma-aldrich Inc. Indirubin-3'-monoxime-5-sulfonic acid (402088), iso-olomoucine (495622), WHI-P180 (681500) were purchased from Calbiochem Inc. The CDK inhibitor, flavopiridol was a kind gift from Dr. Ajit Kumar at the GWUMC. The NF-κB inhibitors included BMS-345541 (401480), SC-514 (401479) were purchased from Calbiochem Inc. and 5-Aminosalicylic acid (430-110-G005), BAY 11-7082 (270-219-M010), BAY 11-7085 (270-220-M010), caffeic acid phenylethyl ester (270-244-M010), diethylmaleate (280-017-G005), Parthenolide (350-258-M025), pyrrolidinedithiocarbamic acid (400-002-G005) were purchased from Alexis Inc. and QNZ (6-amino-4-(4-phenoxyphenylethylamino)quinazoline (EI-352), Wedelolactone (EI-316) were purchased from Biomol Inc. All inhibitors were prepared in 10 mM stock solution. 2,6-Dichloropurine and diethylmaleate were dissolved in ethanol; Flavone was dissolved in acetone; Flavopiridol and pyrrolidinedithiocarbamic acid were dissolved in water; 5-aminosalicylic acid was dissolved in hydrochloric acid. The other twenty-nine inhibitors were all dissolved in DMSO.

### Drugs screening and cell counting

HTLV-1 infected cells and uninfected cells were treated with thirty-five inhibitors at four concentrations including 0.01, 0.1, 1, and 10 μM. Forty-eight hours after treatment, cytotoxicity was primarily determined by the color of media and cell viability by trypan blue exclusion. Cells were counted for the number of living cells every 24–48 hrs. Subsequent focusing experiments used flow data to check for viability and apoptosis.

### Cytoplasmic extracts

Cytoplasmic extracts were prepared according to the following procedure. Briefly, cells were collected and washed with PBS once and then once with 80 μl of ice-cold buffer A (Tris-HCl (pH 7.4, 10 mM), MgCl_2 _(1.5 mM), KCl (10 mM), DTT (1 mM), 0.4% NP-40, phenylmethylsulfonyl fluoride (1 mM), aprotinin (10 μg/ml), pepstatin (1 μM), NaF (50 mM), and Na_3 _VO_4 _(1 mM)). Cells were lysed in 80 μl of buffer A by gently passing the cell suspension through a 28-gauge needle. The cytoplasmic extracts were collected by pelleting for 8 sec in an Eppendorf microcentrifuge and the supernatant was collected. The protein concentration for each preparation was determined with a Bio-Rad protein assay kit (Bio-Rad Laboratories, Hercules, CA, USA).

### Immunoprecipitation and *in vitro *kinase assay

Reaction mixtures (24 μl) contained (final concentrations) 40 mM β-glycerophosphate, pH 7.4, 7.5 mM MgCl2, 7.5 mM EGTA, 5% glycerol, [γ^32^-P]ATP (0.2 mM, 1 μCi), 50 mM NaF, 1 mM orthovanadate, and 0.1% (v/v) β-mercaptoethanol. Phosphorylation reactions were performed with 2 mg of cytoplasmic extract immunoprecipitated with appropriate antibody and washed in lysis buffer containing 50 mM Tris-HCl (pH 7.5), 120 mM NaCl, 5 mM EDTA, 50 mM NaF, 0.2 mM Na_3 _VO_4 _, 1 mM DTT, 0.5% NP-40 and protease inhibitors (Protease inhibitor cocktail tablets, Boehringer Mannheim, one tablet per 50 ml) or with 1 μg of purified recombinant GST-IκBα at 37°C for 1 hour. Reactions were stopped by adding 1 volume of Laemmli sample buffer containing 5% β-mercaptoethanol and ran on a 4–20% SDS/PAGE. Gels were autoradiographed and bands were counted using a Molecular Dynamics PhosphorImager software.

### Immunoblotting

Total cellular extracts (20 μg) were separated by a 4–20% Tris-glycine gel then transferred to a PVDF membrane (Immobilon-P transfer membranes; Millipore Corp.) Following the transfer, the blots were blocked with 5% non-fat dry milk in PBS + 0.1% Tween-20 for 2 hr and washed three times with PBS + 0.1% Tween-20 at 4°C. The blots were then probed with 1:200 dilution of primary antibody against caspase-3 (H-277; Santa Cruz Biotechnology, sc-7148), PARP (H-250; Santa Cruz Biotechnology, sc-7150), CDK2 (M2; Santa Cruz Biotechnology, sc-163), cyclin A (H-432; Santa Cruz Biotechnology, sc-751), cyclin E (C-19; Santa Cruz Biotechnology, sc-198), and actin (c-11; Santa Cruz Biotechnology, sc-1615). The blots were then probed with a 1:750 dilution of secondary antibodies for 1 h at 4°C, followed by washes in PBS + 0.1% Tween-20 and detected using SuperSignal West Dura Extended Duration Substrate Kit (Pierce, Rockford, IL, USA).

### HTLV-1 p19 ELISA

MT-2 cells (HTLV-1 infected) were treated with TNF-α (10 ng/ml) for 2 h, washed, and subsequently treated with a specific NF-kB or CDK inhibitor. Media from MT-2 infected cells were centrifuged to pellet the cells, and supernatants were collected and diluted to 1:100 to 1:1,000 in RPMI 1640 prior to ELISA. Seven days later samples were collected and used for p19 gag ELISA. The HTLV-1 p19 core antigen ELISA kit was from Retro-Tek (Cellular Products) and RT/PCR using HTLV-1 specific Tax primers (data not shown).

### ACH transfcetion of cells

Log phase 293 cells were transfected with 20 μg of ACH.pcTax (wild type HTLV-1 clone) using electroporation method. After transfection, the cells were cultured in complete medium supplemented with 10% fetal calf serum (FCS), 2 mM L-glutamine, 50 μg of penicillin/ml, and 50 U of streptomycin/ml. Cell culture supernatants were collected at 4 days post-transfection, and virus particle production was monitored by p19 ELISA as described above. Drug treatment was 6 hrs after transfection of the 293 cells for a total of 150 hrs.

### Flow Cytometry

For cell cycle analysis, cells treated with or without drugs were collected by low speed centrifugation and washed with PBS without Ca^2+ ^and Mg^2+ ^and then fixed with 70% ethanol. For fluorescence-activated cell sorting (FACS) analysis, cells were stained with a mixture of propidium iodide buffer (PBS with Ca^2+ ^and Mg^2+^, 10 μg/ml RNase A, 0.1% Nonidet P-40, and 50 μg/ml propidium iodide) followed by cell sorting analysis. The acquired FACS data were analyzed by ModFit LT software (Verity Software House, Inc.). Cells were washed twice with cold PBS without Ca^2+ ^and Mg^2+^, resuspended in 1× binding buffer (10 mM HEPES-NaOH (pH 7.4), 140 mM NaCl, 2.5 mM CaCl_2 _) and 5 μl of propidium iodide/10^5 ^cells, and incubated at room temperature for 15 min. Cells were acquired and analyzed using CELLQuest software (BD Biosciences).

Detection of apoptosis through annexin V and PI staining was done according to the manufacturers protocol (BD Pharmingen, San Jose, CA). In brief, cells were washed three times in PBS and re-suspended in binding buffer at 1 × 106 cells/ml. An aliquot of 1 × 105 cells was stained with annexin V-FITC and PI for 15 minutes at room temperature. Analysis was performed on a BD FacsCalibur flow cytometer. Cells were considered to be early apoptotic if they exhibited staining for annexin V, but not PI. The double positive population was considered to be in the late stage of apoptosis.

## Competing interests

The authors declare that they have no competing interests.

## Authors' contributions

EA performed the initial drug screening assays along with WIY, WIY carried most of the subsequent confirmation and Western blots, RB, ZK, and CP carried out confirmatory experiments on Westerns, FACS, as well as kinase assays, KKH and WW provided the day to day leadership and direction for the project, FK also provided the overall direction and the funding for the project.
